# Patient and public perspectives on the availability of their health and advance care planning information to support care at the end of life: a mixed-methods questionnaire study

**DOI:** 10.1136/bmjopen-2024-092353

**Published:** 2026-01-07

**Authors:** Jacqueline Birtwistle, Amy M Russell, Samuel David Relton, Hannah Easdown, Usha Grieve, Matthew Allsop

**Affiliations:** 1 Academic Unit of Palliative Care, University of Leeds Leeds Institute of Health Sciences, Leeds, West Yorkshire, UK; 2 University of Leeds Leeds Institute of Health Sciences, Leeds, West Yorkshire, UK; 3 Compassion in Dying, London, UK

**Keywords:** Patients, Caregivers, Digital Technology, Surveys and Questionnaires, Electronic Health Records

## Abstract

**Abstract:**

**Objective:**

To explore patient and public views and experiences of health professional access to patient health records and advance care planning information to support care at the end of life.

**Design:**

A cross-sectional national online survey of patients and the public using a convergent-parallel approach.

**Setting:**

The survey was distributed across the UK by Compassion in Dying and promoted via newsletters and social media channels of the Professional Records Standards Body and NHS England’s digital workstream network. These partners were purposively selected for their active involvement in end-of-life care, including hospices, clinicians and related charities.

**Participants:**

A total of 1728 participants from 103 UK counties responded, including people with a terminal condition (n=33), with long-term condition (n=442), who provide or have provided care to a person with a long-term or terminal illness (n=229) and who identified as healthy and interested in planning for the future (n=1024).

**Measures:**

Both quantitative data (multiple-choice responses and numerical ratings) and qualitative data (open-ended comments) asking about experiences and views of access to their health and advance care planning information to support their care at the end of life.

**Results:**

Confidence that recorded care preferences would be accessed when needed was low for carers (median=2, IQR 1–4) and moderate for patients (median=3, IQR 1–4). Four themes derived from free-text responses included (1) experience of sharing health information; (2) preparation, communication and understanding; (3) concerns, unknowns and assurance seeking; and (4) preserving dignity and respect: understanding individual contexts.

**Conclusions:**

Respondents acknowledged the opportunity for digital systems to enable access to health and advance care planning information but expressed doubts that professionals would retrieve it when needed, citing past failures. Confidence in record accuracy could be strengthened by patient and carer access. Future research should examine whether such access improves alignment of care with patients’ wishes.

**STRENGTHS AND LIMITATIONS OF THIS STUDY**

- First large-scale national survey exploring patient and carer perspectives on documenting and sharing personal health information and advance care planning information to support end-of-life care.
- Despite being unable to calculate response rates due to the online format, the survey achieved a substantial number of responses.
- High participation from carers provided valuable, experience-based commentary on end-of-life care.
- Limited representation of terminally ill patients: fewer responses from individuals with terminal conditions may have skewed the balance of perspectives.
- Lack of demographic and health data: to maintain anonymity and brevity, no personal or health-related information was collected, limiting the ability to analyse respondent characteristics.

## Background

 Advance care planning involves supporting people in understanding and documenting their values, wishes and preferences regarding future medical care.^1^ The effectiveness of documentation of advance care planning information depends on several factors: patients’ ability to articulate their values, clinicians’ capacity to elicit and record these preferences, and the healthcare service’s ability to access and honour them.^2^ In many services, patients receiving palliative care may interact with multiple professionals across diverse settings, including general practice, hospitals, emergency services and residential aged care.^3^ To support continuity and coordination of care, documentation of preferences must be accessible, updatable and actionable across these settings.^4^

Increasingly, digital systems play a critical role in enabling effective advance care planning across complex healthcare settings.^4^ These systems are designed to facilitate efficient information sharing, reduce duplication and improve timely access to patient records, functions that are essential when multiple professionals across diverse settings are involved in a patient’s care.^4^,^6^ Digital platforms may take the form of structured templates within electronic health records or standalone systems linked to these health records.^6^ When implemented effectively, they allow clinicians across settings to view, interpret and honour patients’ documented wishes, ensuring that care decisions are informed by the individual’s expressed preferences.^7^,^9^ However, there are known challenges with the implementation of digital systems in some countries, such as the UK, where variations and interoperability issues among multiple electronic health record systems hinder effective information sharing. These challenges are compounded by the continued use of both electronic and paper-based approaches in parallel.^10^,^12^

Alongside digital platforms for information sharing by health professionals, online resources are increasingly being used to support advance care planning decisions and documentation, without direct input from a health professional.^13^ Patients often complete these independently or with an advocacy service.^13^ These resources typically require the download of the patient-completed form that must be taken to a health professional to be replicated or a copy made and uploaded to a digital health record.^14 15^ These resources have been shown to increase engagement with the advance care planning process but are standalone documents and not linked with digital systems used by health professionals.^16^

Claims that digital systems improve patient outcomes are difficult to verify, as much of the existing research has focused on quantification of documentation (eg, count of patients with a registered advance care plan, death in documented preferred place of care, ambulance usage)^1017^,^20^ regardless of current relevance to the patient. While a growing number of studies have explored the patient experience of digital sharing of medical records, for example, Benjamins *et al*,^21^ Zanaboni *et al*,^22^ Hägglund and Scandurra^23^ and Kuusisto *et al*,^24^ there remains a notable gap in research examining how patients experience the digital sharing of advance care planning information. A recent study exploring the experiences and views of patients, including those receiving palliative care, found that very few had any direct experience with the digital sharing of advance care planning information.^25^ A crucial missing element in the current evidence base is the voice of patients and caregivers, who have been largely excluded from the design and development of digital systems intended to support the sharing of advance care plans. Their perspectives are essential to ensuring that these systems are not only clinically effective but also meaningful, accessible and responsive to the needs of those they are designed to serve.

This research aimed to explore patient and public experiences and views on sharing information about their health and advance care planning details. Specifically, we sought to understand perceptions of how this information is accessed by health professionals to support care at the end of life, and the extent to which different access features might influence confidence that their information will be used when needed.

## Methods

### Design

A data analysis of an online survey using a convergent-parallel mixed-method design.^26^ This involved the simultaneous collection of both quantitative data (multiple-choice responses and numerical ratings) and qualitative data (open-ended comments), with both types treated as equally important. This approach enabled a comprehensive understanding of the topic, with quantitative data identifying trends and qualitative responses providing context and depth to explain those patterns.

We report the study in accordance with the Checklist for Reporting Results of Internet E-Surveys guideline for online survey distribution and reporting.^27^

### Setting

As part of a public consultation on developing standards for health and care records, Compassion in Dying and the Professional Record Standards Body conducted a national survey to gather views from patients, carers and members of the public across the UK. The survey explored perspectives and experiences regarding health professionals’ access to information about patients’ health (medical information) and their wishes and preferences (advance care planning information), to support end-of-life care. It also sought feedback on potential features of information access, including digital systems that could facilitate such care. This study represents the first comprehensive analysis and reporting of this survey data.

### Participants

A convenience sample was used. Individuals were eligible if they self-identified as having a terminal condition, having a long-term condition, being a current or bereaved carer of a person with a terminal or long-term condition, or a healthy person interested in planning for their future care.

### Data collection

Data were collected in February 2021 using Survey Monkey (http://www.surveymonkey.com). The survey link was distributed via email by Compassion in Dying, using a historic list of approximately 18 500 subscribers who had signed up for support and updates since 2016, acknowledging that some individuals may no longer be alive or remain engaged. No reminder emails were sent. The survey was also advertised in the Professional Records Standards Body newsletter (circulation around 2850) and social media (Twitter/X followers around 2750). NHS England encouraged members of its digital workstream group, including hospices, clinicians and end-of-life charities, to share the survey link with their networks. The survey remained open for 13 days. An a priori sample size calculation was not performed because the study aimed to include all available participants within the study period and was exploratory in nature. Anonymised data were transferred from Compassion in Dying to the researchers using encrypted software. Participants were free to choose whether to complete the survey.

### Survey instrument

The questionnaire was developed and tested for face validity and readability in collaboration with patients and carers. Feedback focused on language clarity and sensitivity around end-of-life topics, and questions were adapted accordingly. They also suggested the inclusion of questions about access to medical information as well as advance care planning information to add context. The survey included four core questions exploring experiences and views on health professionals’ access to documented information about respondents’ health and their wishes and preferences for care at the end of life. Participants were asked to consider scenarios where they or a loved one might become more ill and require care in a different setting. Free-text comment boxes allowed qualitative input (see online supplemental file 1). Demographic information included the role of the respondent and county of residence.

### Data analysis

#### Quantitative analysis

Descriptive statistics were used to summarise survey responses. Medians and IQRs were calculated for two key questions:

- Please rate how confident you feel about the following statements (Webpage 2: Relating to *health professionals’ ability to access their health records, wishes and preferences and people they want involved*).
- What would give you the most confidence that you or a loved one would get the care that’s right for you/ them at the end of life? (Webpage 3: Relating to *features of access to their health records and preferences that would increase confidence in receiving appropriate end-of-life care*)

Counts and proportions were calculated for responses to multiple-choice questions concerning permission to access health records and wishes and preferences (see online supplemental file 1: Webpage 4 (Health Records) and Webpage 5 (Wishes and Preferences)). Respondents were asked to indicate how health professionals should access their records. The options provided were (1) that health professionals should have access whenever it is needed without requiring explicit permission or (2) that they should only access it with permission that is requested once only. Respondents could alternatively select neither of these options and describe their preference for health professional access in an optional text box. These comments were analysed using conventional content analysis,^28^ categorised according to the reasons provided, and summarised descriptively. Findings were presented alongside the corresponding questions.

A Mann-Whitney U test was conducted to explore whether respondents’ level of confidence in professionals accessing their records was associated with their likelihood of providing a free-text comment.

#### Qualitative analysis

Braun and Clarke’s reflexive approach to qualitative analysis was used^29^ to analyse comments in text boxes:

- If there’s something else in relation to your end-of-life care record that is important to you, please tell us here. (Webpage 3)
- If you’ve had a specific experience, or have thoughts about your wishes being known at the end of life, could you tell us a bit more about it? (Webpage 6)

This method is appropriate for analysing surveys that are designed to explore very sensitive topics or when the population is dispersed or difficult to access. Additionally, the specific focus of the survey helped mitigate potential challenges associated with the large sample size, making this analytical approach well suited to the study.

All qualitative data were imported into Microsoft Excel to support initial organisation and coding. Excel was used to structure the data by respondent, enabling efficient sorting, filtering and annotation throughout the analysis process. Responses were treated as a single, unified dataset per participant, allowing for a holistic interpretation of each individual’s viewpoint.

An inductive thematic analysis was employed to identify patterns and themes within the data. This approach enabled the researchers to move beyond the structured survey items and capture the diversity and complexity of participants’ experiences and views.

One researcher (JB) began the analysis by familiarising herself with the full set of responses from each participant. This supported a nuanced understanding of the data and informed the development of preliminary codes. Coding was conducted manually, with attention to recurring ideas and concepts across responses.

Theme development was iterative and collaborative. JB generated initial codes and themes, which were then reviewed and refined in consultation with two additional researchers (AMR and MA). Through a process of discussion and consensus building, the team ensured that the final themes were both representative of the data and analytically robust.

### Patient and public involvement statement

Patient and public representatives engaged in NHS England’s strategic programmes were consulted during the design of the survey instrument. This included individuals receiving palliative care and their carers. Feedback was obtained on language, clarity and the potential impact of items addressing end-of-life preferences, and the questionnaire was revised accordingly.

## Results

There were a total of 1728 responses from eligible participants from most UK counties (103/109; 94.5%) including people with a terminal condition (n=33), people with a long-term condition (n=442), people who provide or have provided care to a person with a long-term or terminal illness (n=229) and people who identified as healthy and interested in planning for the future or offered other reasons for completing the survey (typically a mild condition or a carer for someone with one) (n=1024). Four of these people with a terminal illness and 70 with a long-term condition also provide or have provided care to a loved one with a long-term or terminal illness. Missing data were low at <2% for most questions. All medians are calculated from responses provided (ie, excluding missing data).

### Confidence that the healthcare team will have access to the information they need to support or treat the patient at the end of life

Respondents’ ratings of confidence that the healthcare team supporting or treating them will have access to information about them when caring for them (or the person they care for) at the end of life are shown in table 1. Median ratings indicated a moderate level of confidence that health professionals will access their end-of-life information; this was similar across respondent role groups and the type of information accessed.

**Table 1:** Median ratings of level of confidence in health professional access to patient information at the end of life

Please rate how confident you feel about the following statements (1 to 5 scale, where 1=not confident at all and 5=very confident):				
	Terminal condition (N=33)	Long-term condition (N=442)	Carer (N=229)	Healthy or other (N=1024)
	Mdn (IQR) n	Mdn (IQR) n	Mdn (IQR) n	Mdn (IQR) n
The healthcare team supporting or treating me or a loved one will have access to:				
Information about my health that they need when caring for me at the end of life	3 (1–4) 30	3 (1–4) 386	3 (1–4) 214	3 (2–4) 871
My end-of-life wishes and preferences	3 (1–4) 26	3 (1–4) 380	2 (1–4) 208	3 (1–4) 879
Information on who I want to be involved in decisions about my health and care at the end of life	3 (1–4) 25	3 (1–4) 387	2 (1–4) 213	3 (1–4) 885

Medians calculated as those who provided a rating (ie, discounting missing data and respondents who answered “Don’t know”).

### Priorities for increasing confidence that the healthcare team will have access to the information they need to support or treat the patient at the end of life

Respondents were asked to rate features of access to their health records and documented preferences that they believed would increase their confidence in receiving appropriate end-of-life care (table 2). Ratings were similar across participant role groups and items. Median ratings indicated the most highly rated feature was that health professionals have access to preferences, including the details of the people they want to be involved in care decisions and preferences, such as the place of care and treatments they do and do not want. There was also a high level of importance placed on the ability for patients to access their records via a website or app with the ability to view, edit and share with family.

**Table 2:** Median ratings of confidence in features to support health professionals’ provision of end-of-life care

Please rate what would give you the most confidence that you or a loved one would get the care that’s right for you/ them at the end of life (1 to 5 scale, where 1=this doesn’t matter to me and 5=this is very important to me)				
	Terminal condition (N=33)	Long-term condition (N=442)	Carer (N=229)	Healthy or other (N=1024)
	Mdn (IQR) n	Mdn (IQR) n	Mdn (IQR) n	Mdn (IQR) n
The healthcare team supporting or treating me can see:				
Details of the people I want to be involved in decisions about my care	*5 (5–5)* *32*	5 (5–5) 429	5 (4–5) 225	5 (5–5) 1000
My preferences such as where I want to be cared for	5 (5–5) 31	5 (5–5) 431	5 (4–5) 224	5 (5–5) 998
Which treatments I do and do not want	5 (5–5) 33	5 (5–5) 433	5 (5–5) 225	5 (5–5) 1003
I can:				
View my end-of-life care record via a website or app	5 (4–5) 29	5 (4–5) 401	5 (4–5) 208	5 (4–5) 950
Record and make changes to my end-of-life care preferences via a website or app	5 (4–5) 30	5 (4–5) 400	5 (4–5) 207	5 (4–5) 962
Share my end-of-life care record with my family and loved ones	5 (5–5) 29	5 (5–5) 424	5 (5–5) 216	5 (5–5) 995

Medians calculated as those who provided a rating (ie, discounting missing data and respondents who answered “Don’t know”).

### Preferences for sharing records with health professionals

**Figure 1:**
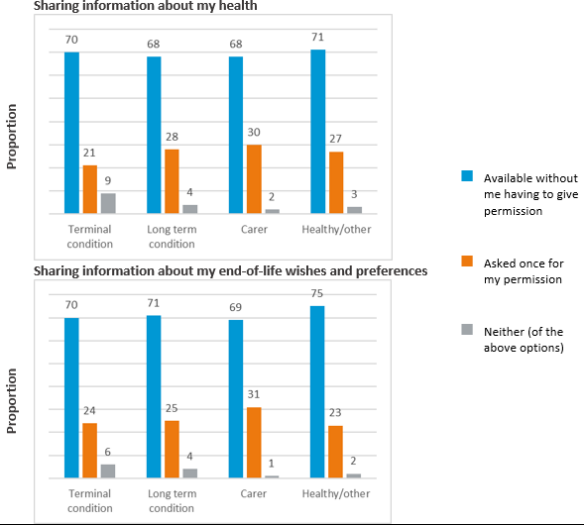
Preferences for sharing information with health professionals. Percentages calculated as those who responded (ie, discounting missing data).

The majority of respondents (68–75% across all role groups) said they would like information about their health and their wishes and preferences to be available to any healthcare professional supporting them whenever they need it, without having to give their permission for this information to be shared (figure 1). A further 23–31% said they would prefer to be asked once for permission. Respondents who chose neither of the above options were invited to provide additional details on their views on access to their information. Conventional content analysis of 82 comments indicated other requirements for sharing information. These included that permission should be sought from the next of kin at the point of need and should be situation-specific or service-specific only (eg, permission-free access for emergency situations or health professionals already known to the person).

### Qualitative analysis

Of the 1726 survey participants, 1071 provided a free-text response in at least one comment box addressing views and experiences. These included prompts such as “If there’s something else in relation to your end-of-life care record that is important to you”, and “If you’ve had a specific experience, or have thoughts about your wishes being known at the end of life”. Comments from participants in the healthy/other group (n=556) were excluded from the free-text analysis to focus specifically on individuals with a terminal illness (TI), long-term condition (LTC) or carers. An additional 69 responses were excluded as they did not relate to experiences or views about documentation, sharing or access to their health or advance care planning information (terminal condition: n=3; long-term condition: n=44; carers: n=22). The final number of respondents whose comments were coded included those with a terminal condition (n=21), those with a long-term condition (n=266) and carers (n=181).

Previous studies exploring patient experience have found that individuals who are more likely to leave a comment tend to report a negative event or viewpoint, and their comments are typically more detailed than those reporting positive experiences.^30^ In the present study, a Mann-Whitney U test revealed that respondents who left at least one comment reported significantly lower levels of confidence that the healthcare team supporting or treating them could access their information, compared with those who left no comment. This was true for all types of information: information about my health that they need when caring for me at the end of life (U=50 548.0, p=0.0171); my end-of-life wishes and preferences (U=48 481.5, p=0.010) and information on who I want to be involved in decisions about my health and care at the end of life (U=52 302.5, p=0.0005).

**Figure 2:**
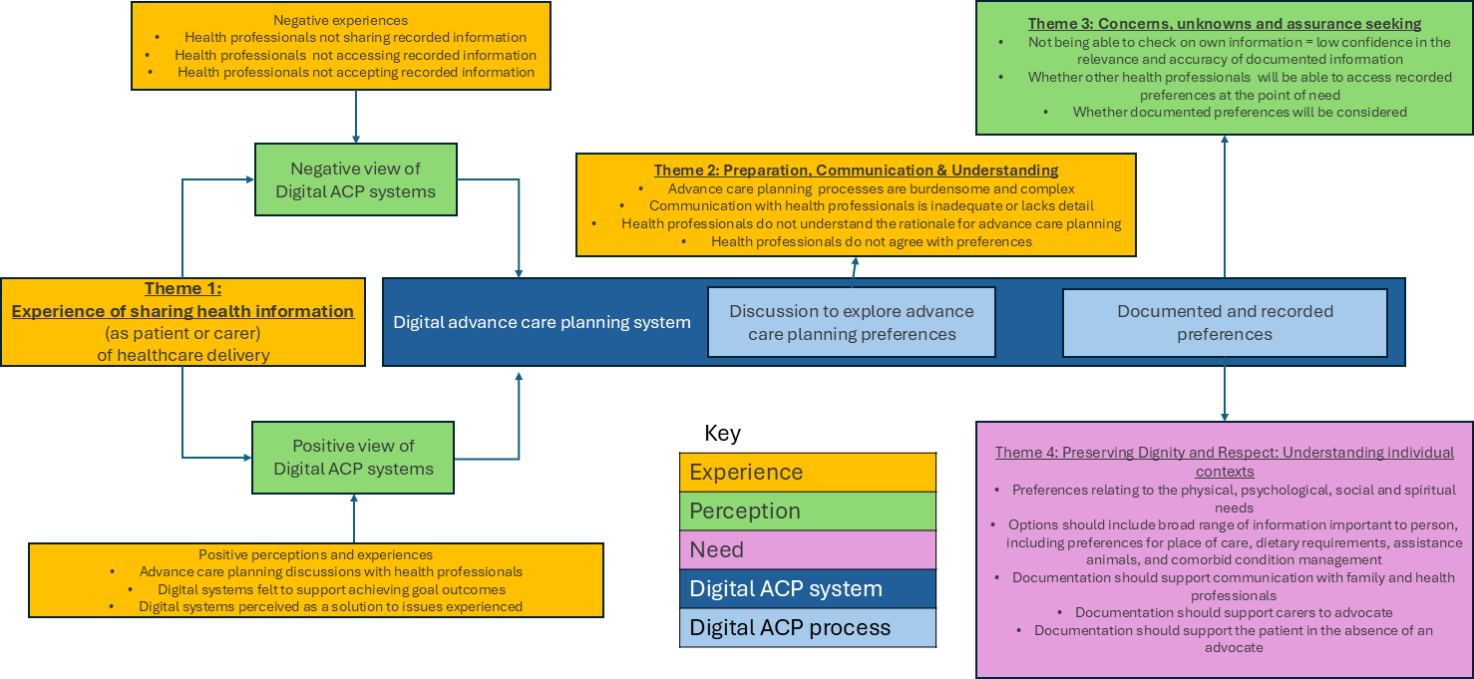
Conceptual map of digital advance care planning (ACP) systems for documentation and sharing of wishes and preferences.

Participants in all groups often spoke from the viewpoint of both carer and patient (eg, patients also spoke about experiences as a carer; carers often considered their own end-of-life care as well as the person they care for). Four themes illustrate how people experienced sharing their health and advance care planning information, and how they articulated their needs related to discussing, documenting, and how this information is accessed and used by health professionals: (1) Experience of sharing health information; (2) Preparation, Communication and Understanding; (iii) Concerns, unknowns and assurance seeking; (4) Preserving Dignity and Respect: Understanding individual contexts. The conceptual map (figure 2) provides a detailed overview of digital systems for documentation and sharing of wishes and preferences. It shows emerging themes that describe how the recording process and the system interact with patients’ needs and concerns and perceptions.

#### Theme 1: Experience of sharing health information

Prior experience of sharing medical or advance care planning information, whether it was digitally transmitted or via other means, shaped participants’ views about how digital records could support their needs at the end of life. Experiences reported included observations relating to the care of another person as well as participants’ own experiences.

Respondents who talked about the perceived benefits of digitally recorded advance care plans had either experienced a positive encounter related to discussion, documentation or sharing end-of-life wishes or had reported an issue. Access to a digital system was perceived as a solution to issues experienced with sharing patient wishes within organisations (Carer 815) and between them (Carer 321).

Every time I go to hospital with my elderly mother, they lose her advanced directive or fail to pass it from one ward to another. A recognised electronic form would be fantastic and very reassuring. Carer 815

I am aware that her DNAR requests are logged with her GP and should be also with the hospital but it seems this is not shared with the hospital. To have the DNAR available online with access allowed would be a benefit. Carer 321

They perceived that health professionals are working together to plan, document and share patient information and that they would access patients’ end-of-life wishes at the point of need.

Easy to have a conversation with my GP about my wishes. She recorded my wishes on the computer system, and has contacted me every 6 months to ensure there is no amendment. LTC 828

Conversely, participants who held a negative perception of documentation or sharing of their information had often experienced instances where health professionals had not accessed their information or had been reluctant to accept or action it.

I am in my 30s and have a DNACPR, RESPECT form and Advanced Directive in place due to serious health complications. I have often had Ambulance staff and hospital staff not believe my story. LTC 163

#### Theme 2: Preparation, communication and understanding

Respondents were keen to document advance care planning information so it would be available to health professionals. However, they considered this to be a burdensome, complex process they would have to complete alone or with inexperienced health professionals.

I would really like to crack on and get this sorted, H&W PoA, Advance Directives etc. but the bureaucracy is daunting. LTC 1525

I have completed the Coordinate My Care process, which shares info from GP with the ambulance service. It is not simple yet rather limited, basically DNAR? and do you want to die at home? My GP had never done this before. LTC 444

Communication about what advance care planning involves was often inadequate and lacked detailed information about the process that the patient or family could understand. This led to confusion for carers about the purpose of this discussion and a lack of confidence in health professionals’ understanding of their family members’ wishes.

GP offered a discussion about DNACPR and ceilings of treatment by sending a post-it note home with a family member after a routine appointment. Family members didn’t know what this was. Not well explained. …. Not really confident in GP’s understanding of Dad’s wishes. Carer 142

They reported discrepancies between their own and health professionals’ understanding of their rationale for documenting their preferences, as well as the decisions they have made, and often felt their ideas and preferences would not be accepted by health professionals and therefore ignored.

I have given my GP my ACP, but she seemed perplexed as to why I was doing this since I was not ill. I am not convinced they really get the importance of doing this early, basically because I think many medical workers are still stuck with the idea that their job is about curing us, not helping us to live well. LTC 671

#### Theme 3: Concerns, unknowns and assurance seeking

Respondents expressed concerns about health professional interaction with their information at each stage of digital documentation of preferences. They raised concerns about the level of accuracy of the information recorded about them, and they reported times when health professionals could not easily access their information. Additionally, they were unable to check their own information, resulting in little confidence that it would be accurate and relevant. The following quote describes the person’s perception of the use of paper versus digital records for their data and the unmet need for the patient to check the information recorded.

Many professionals have been involved in my relative’s care. Most bring & write on paper. Where laptops are used the information seem unstructured, with clinicians having trouble finding the information they or my relative needs. Much of the care feels disjointed … Not once have records been shared with my relative, for checking detail or accuracy. Carer 1727

As well as concerns about documentation, respondents also questioned whether other health professionals could access their record at the point of need. Some raised concerns about their information being accessed by parties to which they had not granted permission.

I have had my healthcare record shared with an insurance company by my gp without my permission … I have family members who do not know of my diagnosis and I do not wish [them] to know, so am worried that if this was a public message on my medical records it would be released without my permission. LTC 194

They were also aware that the existence of a digitally documented record did not mean health professionals would access it, and if they did that, they would provide care in line with documented wishes and preferences.

My wife and I have registered our preferences / requirements with our GP and our local hospital, but we are both concerned that the information will be overlooked when the time arrives because hospital staff and particularly GP staff appear to have very poor understanding / knowledge of such advanced decisions. TI 1074

#### Theme 4: Preserving dignity and respect: understanding individual contexts

Respondents described the type of information they considered important to document to inform their future care. Preferences that preserve dignity and respect were favoured over life-preserving medical interventions. It was particularly important to them that physical, psychological, social and spiritual needs are understood by family as well as health professionals. The type of preferences reported included the preferred place of care, remaining with assistance animals, dietary needs and how their specific health conditions should be managed. They considered the importance of stating treatments not yet available (such as a medically assisted death) in the case of these becoming a possibility.

I have a spinal cord injury which requires very specialist care to manage bladder, bowels, pressure sore preventions, spasms, muscle contractions if legs are not stretched. I am extremely anxious as I currently manage my own care as GPs and local hospitals have virtually no knowledge … I will definitely want appropriate care to end my life with dignity. LTC 1202

Patients were aware of potential discrepancies between family members’ perceptions of how they should be cared for. They expressed concerns that future treatments and care may not align with stated wishes and preferences in families with different views. They saw a role for the digital record in guiding appropriate care by providing evidence of the patient’s preferences to family members.

I had a major surgery so filled in an electronic record and printed it off … We have done the same for my elderly mother … the fact we have them makes me confident that things will go both how my mother wishes (my siblings were given copies of hers so there can be no arguments) and my wishes will be carried out. LTC 593

Participants talked about self-management of their health condition. Comments reflected their anxiety that information necessary to be able to do this in a way that preserves dignity, respect and comfort would not be available to health professionals looking after them. Digital systems were perceived as a means of communicating information about preferred and holistic methods of symptom management, should the patient not be able to communicate this themselves.

I worry about the practical care in hospital because of the daily pain I have; in the event I am not able to communicate to my caregivers I fear being manhandled. LTC 1509

They welcomed a documented plan that is accessible to health professionals, and they envisaged it would support having their wishes respected, either through supporting carers in advocating for patients or to be a substitute for advocacy for those with no next of kin.

I personally am worried that my next of kin is not strong enough to advocate for me at end of life. I am therefore determined to document my wishes clearly to reduce risk of wishes not being followed through lack of courage or confidence. Carer 941

## Discussion

This large-scale survey of patients and the public captured experiences and views of how shared patient records support patients receiving palliative and end-of-life care. Respondents were in favour of their recorded information being shared across all settings yet had only had moderate levels of confidence that it would be accessed or acted upon. This reflects a gap between the potential of record sharing and its perceived reliability in practice.

Participants valued being able to specify who should be involved in care decisions, as well as digital features such as access via a website or app that allow them to view, edit and share records with family members. Perceptions of sharing information were shaped by personal or observed experiences of care delivery. Where participants had positive experiences with information sharing, digital systems were seen as beneficial. Conversely, those who had encountered breakdowns in communication viewed digital tools as a potential solution to avoid similar issues in the future.

Low confidence that health professionals will read patient advance care planning information is consistent with previous research showing that hospital-based clinicians rarely consult information recorded by community-based professionals.^31^ Concerns about where personal information is stored, who can access it, and its accuracy echo findings from UK-based studies.^32^ For example, Caine *et al*^33^ found that patients were often unaware of how widely their health information was shared, and half did not know what data were stored. When looking at earlier work by Geerse and colleagues, advance care planning documentation was fully concordant with conversations between patients and clinicians only 43% of the time.^34^ This is against the backdrop of general mistrust in medical records for patients—Li *et al*, for example, like the findings of this research, highlighted that most people can describe how poor health information sharing within EHRs has negatively impacted care.^35^

In the present study, participants valued the potential to access their digital records. This could support caregivers in decision-making, particularly given evidence of variability in predicting the end-of-life preferences of the person they care for.^36 37^ Furthermore, our findings suggest that by also allowing carers to access up-to-date digital records may empower them when making decisions on behalf of patients. This may also help mitigate family pressure to continue treatment and the emotional impact of grief, which can disrupt care aligned with patient preferences.^37^ Our findings suggest that by accessing up-to-date patient information via a shared digital record, carers may feel supported when asked to make end-of-life decisions on behalf of the patient.

Importantly, participants emphasised the need for digital systems to reflect values such as dignity and respect, rather than solely focusing on clinical outcomes or life extension. This resonates with prior research highlighting the importance of treatment preferences grounded in self-dignity and personal values.^37 38^ Participants also expressed concern that their choices might be questioned or rejected if not understood, emphasising the need for systems that support clear communication and shared understanding.

Freely available digital resources that allow patients to document personal values and preferences, particularly those that can be printed and shared, may offer reassurance in cases of disagreement (eg, MyWishes.co.uk^15^ and Hospice UK^39^). Implemented well, online advance care planning tools can be practical and feasible.^1640^,^43^ To support individual choice and trust, systems should include features such as easy patient access to records, visibility of sharing permissions, control over access, contextual privacy settings and notifications when information is viewed.^20^

### Strengths and limitations

To our knowledge, this study represents the first large-scale national survey exploring patient and carer perspectives on documenting and sharing advance care planning information to support end-of-life care. The survey achieved a substantial number of responses and high participation from carers provided valuable, experience-based insights on end-of-life care.

Several limitations should be noted. The survey was distributed to individuals on a register maintained by Compassion in Dying, which included people who had previously received support or consented to receive updates. It is likely that some individuals were no longer alive or were carers and family members whose circumstances and interest in the organisation had changed over time. Consequently, the denominator for potential respondents was uncertain, making it impossible to calculate an accurate response rate. In addition, the use of a convenience sample and online distribution may have introduced selection bias. Recruiting participants through organisations that support end-of-life care and allowing self-selection likely resulted in a sample with greater interest, awareness or experience in end-of-life care than the general population. Although the survey included individuals with a terminal condition, their representation was relatively low compared with other groups. Additionally, to preserve anonymity and keep the survey brief, no demographic or health-related information was collected. This approach maximised participation and allowed for a wide range of experiences to be captured, but it limited the ability to assess representativeness or analyse respondent characteristics.

Importantly, the qualitative component of this study was not intended to produce generalisable findings. Rather, it aimed to provide rich, contextual insights into participants’ experiences and views. The depth of qualitative data offers valuable understanding of how digital systems for sharing advance care planning information are perceived, even if these insights may not be broadly representative of all patient or caregiver populations.

In common with other large-scale cross-sectional healthcare experience surveys, negative experiences were more detailed and specific, providing richer material than positive experiences.^30 44^ As a result, we may have missed out on detailed information about positive experiences and views of how digital systems are meeting patient needs.

## Conclusion

This study highlights the importance of incorporating patient and caregiver perspectives into the design and implementation of digital systems for sharing their information. Doing so can help ensure that these tools are not only technically functional but also aligned with the values and needs of those they are intended to serve. While patients and carers report the potential for digital systems to facilitate documentation and sharing of their information, they also question how these systems will support care in reality, and how accessible they are to patients once created.

Efforts should be made to build confidence and clarify the expectations of patients and members of the public around the documentation of their wishes and preferences for care alongside the subsequent sharing and use of this information. Furthermore, future research is required to explore whether such patient and carer access to their record influences confidence in the accuracy of content and the likelihood of care being delivered in line with their wishes.

## Supplementary material

10.1136/bmjopen-2024-092353 online supplemental file 1

## Data Availability

Data are available upon reasonable request.
