# Supplementary material for: Patient and public perspectives on the availability of their health and advance care planning information to support care at the end of life: a mixed-methods questionnaire study
Source: BMJ Open. 2026 Jan 7;16(1):e092353. doi: 10.1136/bmjopen-2024-092353 (PMC12781988; doi:10.1136/bmjopen-2024-092353)
Supplement: Supplementary file 2 [file bmjopen-2024-092353.reviewer_comments.pdf]

PEER REVIEW HISTORY

BMJ Open publishes all reviews undertaken for accepted manuscripts. Reviewers are asked to complete a checklist review form and are provided with free text boxes to elaborate on their assessment. These free text comments are reproduced below.

ARTICLE DETAILS

Title (Provisional)

Patient and public perspectives on the availability of their health and advance care planning information to support care at the end of life: A Mixed-Methods Questionnaire Study

Authors

Birtwistle, Jacqueline; Russell, Amy M; Relton, Samuel David; Easdown, Hannah; Grieve, Usha; Allsop, Matthew

---

VERSION 1 - REVIEW

**Reviewer**	**1**
**Name**	**Hosseini, Lida**
**Affiliation**	**Iran University of Medical Sciences, Department of Community Health and Geriatric Nursing**
**Date**	**04-May-2025**
**COI**	**None**

I have reviewed your article entitled: “Patient and public experience and views on digital systems for sharing records for health and care preferences at the end of life”

the subject of this manuscript very important and innovative. However, there are several important points that need to be addressed before considering the paper for publication and I would like to offer some constructive comments:

Introduction

1. This section is written very briefly. It is better to follow the logic of the introduction. It is recommended that this section be enriched and include sub-sections.

- ✓ Expressing the importance of Digital systems and its benefits for individuals, families, and health care systems.
- ✓ It has been noted that the literature on the main concept of this work is limited but lacks credible scientific citations. This issue should be discussed further.
- ✓ A description of the studies that have examined this issue to date in the world and UK.
- ✓ Finally, the existing knowledge gap, the importance of the study topic, and the purpose of this study must be clearly explained.
- ✓ Totally, this part has been written very ambigious, and I didn't understand the importance of the issue and what was going to be done.

2. The purpose of the study is written at the end of the introduction, so it should be removed from the methods section and added to the end of the introduction.
3. The written format of the study design is incorrect. Please correct it.
4. Please more explain about convergent-parallel mixed method design.
5. The data analysis method is not clear. In qualitative analysis, depending on the subject of the study and whether there is a known framework and theory, thematic or... method is used, which is very vague. Explain the methods more clearly.
6. In the methodology section, it is better to specifically mention the study design, methodology, data extraction method, demographic characteristics of the samples, and analysis method separately and specifically. This section is written ambiguously and needs further clarification.
7. Although a complete explanation is provided in the discussion section, its main drawback is the lack of comparison of the findings with other studies, which is the most important part of the discussion related to these comparisons.
8. The application of the findings in future research, national-level planning, and policymaking should be mentioned separately and in a separate section in the end of discussion part.

**Reviewer**	**2**
**Name**	**Holmen, Heidi**

**Affiliation**	**Oslo Metropolitan University**
**Date**	**08-Jul-2025**
**COI**	**None**

Thank you for the opportunity to read and review the submitted manuscript. I find the topic interesting and timely, and the manuscript clear and thorough. I do not have any major comments on the draft, but some minor reflections to perhaps add even more clarity. Best of luck with the manuscript!

1. Consider adding digital to the aim, that the sharing is digital? To add some more context. Or, as the aim in the main manuscript is a broader one, I suggest adding this to the abstract, having two identical aims presented?

2. The abstract and introduction is otherwise sufficiently clear.

3. Consider moving the details on the reporting guide (CHERRIES) to the subheading "design" of the methods.

4. Participants – add any age criteria if relevant – or all ages?

5. Analysis – perhaps adding a rationale for why you explored associations between confidence in healthcare personnel viewing their wishes and adding of comments?

6. The results are presented clearly and the figures add relevant details, particularly the concept map. I cannot find any clear results of the content analysis, or is there a mismatch between the questions in the analysis and results that were subject to content versus thematic analysis?

7. The discussion is addressing the results, drawing on previous research in a nuanced way, with relevant limitations being addressed.

Reference 7 is missing publication year, please ensure all references are correct

VERSION 1 - AUTHOR RESPONSE

Reviewer Point	Response
**Reviewer: 1**	
Introduction	

1. This section is written very briefly. It is better to follow the logic of the introduction. It is recommended that this section be enriched and include sub-sections.<br>a. Expressing the importance of Digital systems and its benefits for individuals, families, and health care systems.<br>b. It has been noted that the literature on the main concept of this work is limited but lacks credible scientific citations. This issue should be discussed further.<br>c. A description of the studies that have examined this issue to date in the world and UK.<br>d. Finally, the existing knowledge gap, the importance of the study topic, and the purpose of this study must be clearly explained.<br>Totally, this part has been written very ambigious, and I didn't understand the importance of the issue and what was going to be done	Thank you for this detailed and constructive feedback.<br>We have revised and substantially expanded the introduction section to follow a clearer logical structure and include sub-sections.<br>Specifically, we have:<br>a. Highlighted the importance of digital systems for individuals, families, and healthcare systems, including their potential benefits.<br>b. Discussed existing literature on the topic with appropriate scientific citations to support the statements.<br>c. Summarised relevant studies to provide context for the study.<br>d. Clearly identified the existing knowledge gap, the significance of the study topic, and the specific purpose of this study.<br>These revisions aim to make the rationale, significance, and objectives of the study much clearer and easier to follow.
2. The purpose of the study is written at the end of the introduction, so it should be removed from the methods section and added to the end of the introduction.	Thank you for this suggestion – we have moved this to the end of the introduction.
3. The written format of the study design is incorrect. Please correct it.	Thank you for this comment. We have corrected the format of the study design in the manuscript to align with the journal's preferred style and guidelines.
4. Please more explain about convergent-parallel mixed method design.	Thank you for this constructive suggestion. We have expanded the description of the convergent-parallel mixed methods design in the Methods section and included a relevant reference to clarify the approach and its application in our study.
5. The data analysis method is not clear. In qualitative analysis, depending on the subject of the study and whether there is a known framework and theory, thematic or… method is used, which is very vague. Explain the methods more clearly.	Thank you for this helpful comment. We have expanded and clarified the description of the qualitative data analysis in the Methods section, specifying the approach used, how themes were developed, and how the analysis was conducted in relation to the study framework. Braun and Clarke's reflexive approach to qualitative analysis in surveys (2020) has been cited. We have clarified that an inductive thematic analysis was conducted on the survey's open-ended responses, describing

	how data were organised in Excel, coding was performed, and themes were developed iteratively and collaboratively to ensure they accurately reflect participants' perspectives.
6. In the methodology section, it is better to specifically mention the study design, methodology, data extraction method, demographic characteristics of the samples, and analysis method separately and specifically. This section is written ambiguously and needs further clarification.	Thank you for this constructive feedback. We have revised the Methods section to clearly and explicitly describe the study design, overall methodology, data extraction procedures, and data analysis methods in separate subsections. We have stated that demographic information included the role of the respondent and county of residence.<br><br>We have also provided more detailed information about the survey questions and comment boxes, instead of referring readers to the supplementary material, to clarify aspects of the survey design and methodology. These changes aim to improve clarity and ensure that all methodological aspects are transparent.
7. Although a complete explanation is provided in the discussion section, its main drawback is the lack of comparison of the findings with other studies, which is the most important part of the discussion related to these comparisons.	Thank you for this comment. We have revised the discussion section to make more explicit links between our findings and existing literature. However, due to the limited number of comparable studies in this area, the scope for direct comparison remains restricted.
8. The application of the findings in future research, national-level planning, and policymaking should be mentioned separately and in a separate section in the end of discussion part.	Thank you for this suggestion. We have added a section at the end of the discussion outlining the potential applications of our findings for future research, national-level planning, and policymaking.
**Reviewer 2**	
1. Consider adding digital to the aim, that the sharing is digital? To add some more context. Or, as the aim in the main manuscript is a broader one, I suggest adding this to the abstract, having two identical aims presented?	Thank you for this suggestion. We have amended the abstract to explicitly include "digital" in the aim, providing additional context while keeping the broader aim in the main manuscript unchanged.
2. The abstract and introduction is otherwise sufficiently clear.	Thank you for this positive feedback. We have further refined the abstract and introduction to incorporate the suggestions from Reviewer 1.
3. Consider moving the details on the reporting guide (CHERRIES) to the subheading "design" of the methods.	Thank you for this suggestion. We have moved the details on the CHERRIES reporting guide to the Design subheading within the Methods section.
4. Participants – add any age criteria if relevant – or all ages?	We don't have this information and have explained that detailed demographic information wasn't collected
5. Analysis – perhaps adding a rationale for why you explored associations between confidence in healthcare	Thank you for this suggestion. We have added the rationale for exploring these associations to the second paragraph of the Qualitative

personnel viewing their wishes and adding of comments?	Analysis section, clarifying the purpose and relevance of this part of the analysis.<br>For clarity we have also added details about the number of comments coded for each participant group and clarified any exclusions, enhancing transparency of the analysis.
6. The results are presented clearly and the figures add relevant details, particularly the concept map. I cannot find any clear results of the content analysis, or is there a mismatch between the questions in the analysis and results that were subject to content versus thematic analysis?	Thank you for this comment. The content analysis is summarised under the heading Preferences for sharing records with health professionals. Specifically, content analysis of 82 comments indicated additional preferences for sharing information, such as seeking permission from next of kin at the point of need or allowing situation-specific access (e.g., emergency situations or known health professionals).<br>These responses were limited in variation and primarily allowed respondents to explain why they chose neither of the fixed options. As such, they were not related to the questions designed to elicit richer qualitative data and were therefore not included in the thematic analysis.
7. The discussion is addressing the results, drawing on previous research in a nuanced way, with relevant limitations being addressed.	Thank you for this positive feedback. We appreciate your recognition of the discussion's clarity and consideration of relevant limitations.
Reference 7 is missing publication year, please ensure all references are correct	Thank you for pointing this out. The missing publication year was for reference 6 (Cevik et al.). We have corrected this and reviewed all references to ensure they are now accurate and complete.
